# The Characteristics of the Growth and the Active Compounds of *Angelica gigas* Nakai in Cultivation Sites

**DOI:** 10.3390/plants9070823

**Published:** 2020-06-30

**Authors:** Yunmi Park, Pil Sun Park, Dae Hui Jeong, Sujin Sim, Nahyun Kim, Hongwoo Park, Kwon Seok Jeon, Yurry Um, Mahn-Jo Kim

**Affiliations:** 1 Department of Forest Bioresources, National Institute of Forest Science, Suwon-si 16631, Korea; otttr@korea.kr; 2 Department of Forest Sciences, Seoul National University, Seoul 08826, Korea; pspark@snu.ac.kr; 3 Forest Medicinal Research Center, National Institute of Forest Science, Yeongju-si 36040, Korea; najdhda@korea.kr (D.H.J.); knh1125@korea.kr (N.K.); redrain39@korea.kr (H.P.); jks2029@korea.kr (K.S.J.); urspower@korea.kr (Y.U.); 4 Forest Biomaterials Research Center, National Institute of Forest Science, Jinju-si 52817, Korea; sujin0606@korea.kr

**Keywords:** *Angelica gigas* Nakai, active compounds, correlation analysis, growth, root

## Abstract

The active compounds of medicinal plants vary in composition and content depending on environmental factors, such as light, temperature, and soil. According to the Korean Pharmacopoeia standards for herbal medicine, the sum of nodakenin, decursin, and decursinolangelate, which are the marker components of Korean *Angelica*, should be at least 6.0 g/100 g. However, the content of the components in Korean *Angelica* cultivated in South Korea often fall below 6.0 g/100 g, due to weather conditions and cultivation site characteristics. This study aimed to gather information about environmental factors that affect the root growth and the content of active compounds. In total, 18 cultivation sites in Pyeongchang, Jecheon, and Bonghwa regions in Korea were investigated for this study. Environmental factors, such as the monthly mean temperature, mean relative humidity, duration of sunshine, total precipitation, soil acidity, and the characteristics of soil nutrient, were investigated over the growing season from April to October 2017. As for the growth characteristics, the dry weight of roots of Korean *Angelica* was measured. The sum of the contents of the three active compounds was 5.3–7.0 g/100 g and the nodakenin content was 0.3–1.3 g/100 g in the cultivation sites. This study concludes that the root yields in the cultivation sites would be improved if weather conditions are maintained with similar levels as those in their natural habitats. Additionally, the environment that improves root growth did not increase the content of active compounds; however, when there was a lot of gravel or high temperatures during the growth period, the content of active compounds was relatively high.

## 1. Introduction

‘Korean *Angelica*’ (*Angelica gigas* Nakai) is a perennial plant of the genus *Umbelliferae*, where young shoots are eaten as seasoned vegetables and roots are used for medicinal purposes. The plant grows to a height of 1 to 2 meters, purple double umbrella-shaped flowers bloom between August and September, and there will be many small rootlets on the thick roots [1]. The origin of the *Angelica gigas* is in East Asia and has been distributed in Korea and northeastern China. However, the confirmed natural habitat on the Korean Peninsula is the cool-temperate mountain areas, which corresponds to mid-to-north highlands and is located in areas adjacent to the valley at 700 to 1300 m [2]. As Korean *Angelica* prefers low-temperature and long-day conditions, the roots are wooded, and the medicinal properties are reduced when the summer depression occurs during high temperatures or when the flower stalks are raised early [3]. Due to the physiological and ecological characteristics available in the highlands, the Korean *Angelica* has been growing in Gangwon Province or in the mountains of North Gyeongsang Province in Korea.

The medicinal properties used by humans are a combination of species-specific secondary metabolites in plants [4]. Unlike primary metabolites, such as carbohydrates, proteins, lipids, chlorophyll, and nucleic acids, which are essential for plants to maintain and increase their cell number, secondary metabolites play an important role in the physiological function of plants. These secondary metabolites not only play an important role in plant chemical defense of species against herbivores [4,5] but also engage in non-biological stresses, such as temperature, moisture, light, and changes in inorganic nutrients. Recently, it has been found to be involved in the regulation of gene expression at the cell level, or signal transmission [6]. Currently, about 300,000 species of plants worldwide are known to produce secondary metabolites. About 1500 types of substances are extracted and separated from plant bodies every year, and about 300 of them are considered as useful materials for biological activity. Most of the plant-generated secondary metabolites are also used to make dyes, polymers, fibers, adhesives, oils, waxes, spices, and perfumes and are useful and valuable in developing new medicines, antibiotics, pesticides, and herbicides [6].

Although ‘Korean *Angelica*’ is mentioned more than 500 times in ‘Donguibogam’ prescriptions and more than 150 times in ‘Bangyakhappyeon’, the plants used in China and Japan are different from the original plant used in Korea. In Korea, the ‘Korean *Angelica*’ (*Angelica gigas* Nakai) is used, while the ‘China *Angelica*’ (*Angelica Sinensis* Oliv. Diels) is used in China, and the ‘Japan *Angelica*’ (*Angelica acutilova*) is used in Japan [7]. Only the ‘Korean *Angelica*’ and ‘Japan *Angelica*’ are grown in Korea, as the climate is not suitable for the growth of ‘China *Angelica*’ [8]. It has been argued that the Korean *Angelica* should be used to distinguish its origin in Korea, China, and Japan because it is of great importance in clinical trials. The main active compounds of the Chinese *Angelicia* are essential oil, organic acids, and polysaccharides, and the major active compounds are z-ligustilide (3-butylylide4, 5-dihydrophthalide), which is a neutral oil, butylidenephthalide, and ferulic acid [9]. The Japanese *Angelica* are generally similar to Chinese *Angelica*, and contain z-ligustilide, n-butylidenephthalide, ferulic acid, and polysaccharide [10,11], but additionally to these, it contains 9,12-octaneacid [12]. The main active compounds of Korean *Angelica* are fat-soluble coumarins, which include decursin, decursinolangelate, umberliferone, nodakenin, peucedanone, marmesin, demethylsuberosin, isoimperatorin etc. Among the compounds, decursin, decursinolangelate, and nodakenin are the major active compounds. Decursin is a natural component of the pyranocoumarin system, which was first separated from the ether extract of *Angelica decursiva* Fr. et Sav. in Japan and has the molecular formula of C_19_H_20_O_5_ with a molecular mass of 328.35 g. The Korean *Angelica*, in particular, contains a large amount of decursin compared with other *Angelicas* [13].

The most active areas of research on the pharmacology of *Angelica gigas* Nakai, *Angelica Sinensis* Oliv. Diels, and the *Angelica acutilova* are on their effects on decreasing the blood pressure, ischemic heart disease treatment, blood clotting delay [14,15,16] effects on the central nervous system, protection against neurotoxicity, and improvement of memory [17,18]. The study of anticancer action is mainly based on studies of decursin and decursinolangelate, which are the main ingredients of Korean *Angelica* [19]. In addition, continuous research has been conducted on areas, such as immunity, anti-inflammatory, and hemorrhage properties [15,20]. Nodakenin is likely to be used as a medicine and health function food for the prevention and treatment of degenerative brain diseases due to its nerve cell protection properties [15]. Nodakenin also inhibits the secretion of allergens in fat cells and showed excellent anti-allergic effects in allergy animal models [15,16]. Most of the Korean *Angelica* cultivation sites today are created by planting seeds taken from their natural habitat; however, these cultivation sites often do not contain optimum conditions needed for the production of increased levels of roots and active compounds. Apart from these adverse conditions, due to the changes in weather conditions also, the amounts of active compounds, which are secondary metabolites, are less than 6 g/100 g as stipulated by the Korean Pharmacopoeia. It is predicted that the number of Korean *Angelica* cultivation sites, which are vulnerable to climate change, will decrease from 12% of the total current cultivation areas to less than 1% by 2040 [21]. The active compounds of medicinal plants vary in composition and content depending on the period of growth and the environment, such as light, temperature, and soil. Korean *Angelica* has been cultivated in sites in various locations and environmental conditions, and its supply and demand as raw material is not smooth due to the fluctuating content of active compounds depending on environmental factors.

Therefore, research on environmental factors that affect the content of active compounds is needed to improve the quality of locally produced Korean *Angelica* as a functional raw material. Until now, no studies have been conducted in which the active compounds of the Korean *Angelica* were analyzed together with the characteristics of the growth in the cultivation site [7,13]. Thus, this study aimed to identify the environmental factors affecting the root growth and content of active compounds in the roots of *Korean Angelicas* grown in the cultivation sites and to establish a database to select optimal cultivation conditions.

## 2. Results

### 2.1. Soil Characteristics

An analysis of the gravel content of the soil in the cultivation sites showed that the content did not show much difference between the cultivation sites in Pyeongchang, and varied between 30.0% and 38.9%. On the other hand, all of the sites in Jecheon showed a high value of over 35% on average, with a large difference in the amount of gravel among the sites. In particular, Je1-3 had the highest amount among the 15 sites with a 62.0% gravel content. In the Bonghwa, Bon-2 had the highest gravel content at 44.4% while Bon-3 had the lowest at 26.0%. Among the 18 sites in all three regions, Pye1-1 had the highest statistically significant clay content, which was at 14.5%, while the Bon-3 and Bon-4 sites had the lowest clay content at 5.0% to 6.4% (Table 1, *p* < 0.05).

The chemical properties of soil were generally high in the order of Pyeongchang, Jecheon, and Bonghwa by region, but they showed various characteristics for each site within the region. In the case of soil acidity, out of a total of 18 cultivation sites, the highest was seen in Je4-2 at 7.7 pH and the lowest was seen in Pye2-5 at 4.2 pH. The content of organic matter, available P_2_O_5_, and the cation exchange capacity (CEC), which are indicators of soil nutrients, were statistically significantly higher in Pyeongchang than in other regions. In the case of cation exchange, out of a total of 18 cultivation sites, the highest K^+^ was seen in Je2-1 at 1.0 cmolc kg^−1^. The highest Na^+^ was in Je1-3 and the lowest Ca^2+^ was seen in Pye2-5. The lowest Mg^2+^ was in Pye2-1 at 0.3cmolc kg^-1^ and the highest in Je3-2 at 2.4 cmolc kg^−1^ (Table 2, *p* < 0.05).

### 2.2. Growth Characteristics

The Pyeongchang region showed the highest average fresh weight of individual roots, which was 467.5 ± 275.6g, while Jecheon area showed the lowest value at 293.9 ± 218.6 g. The root dry weight was 100.4 to 167.9 g in the Pyeongchang area, while in the Jecheon area, an average value of less than 100 g was shown, with a wide variation of 33.5 to 149.4 g between the cultivation sites. According to the characteristics of the cultivation sites, the fresh weight and dry weight of the roots in the Pye1-2 were statistically the highest among the 18 sites in the entire region. In addition, Pye2-5 has the highest fresh weight of a root/shoot growth ratio of 5.3, making the root growth far superior to that of the shoot growth. Je1-1 and Bon-4 both showed statistically the lowest root/shoot ratio among the 18 cultivation sites (Table 3, *p* < 0.05).

Correlation analysis was performed between the root dry weight, water content in root, and root/shoot ratio, which are the main growth characteristics for the cultivation sites, and data obtained from the weather measuring devices installed in each site. As a result, the root dry weight showed a negative correlation with temperature-related factors from April to October, and a positive correlation with altitude and air humidity (Table 4, significant at *p* = 0.01). Additionally, the root/shoot ratio showed a positive correlation with altitude (Table 4, significant at *p* = 0.01). An analysis of the correlation between the soil characteristics of the cultivation sites and root growth characteristics showed that root dry weight showed a positive correlation with the organic matter content (Table 5, significant at *p* = 0.01).

### 2.3. Characteristics of Active Compounds

According to the analysis of active compounds from the four sites in Pyeongchang, nodakenin was at 0.29 to 0.35 g/100 g, decursin was at 2.90 to 3.22 g/100 g, and decursinolangelate was at 2.07 to 2.38 g/100 g, and the total of the three compounds was 5.63 g/100 g on average (Table 6). The Jecheon area had 0.76 to 1.27 g/100 g nodakenin, 2.49 to 4.42 g/100 g decursin, and 2.03 to 3.43 g/100 g decursinolangelate, which was higher than that of the Pyeongchang sites, and showed an average of 7.02 g/100 g for the total of three components (Table 6). In the Bonghwa region, the value of nodakenin was 0.64 to 0.88 g/100 g, decursin 3.22 to 3.46 g/100 g, decursinolangelate 1.93 to 2.37 g/100 g, and the total content was 6.17 g/100 g on average (Table 6). Thus, there was a difference in the content between regions, decreasing in value in the order of Jecheon, Bonghwa, and Pyeongchang, and overall, there was a tendency to have a high content of active compounds in sites with a low average value of root dry weight. Je1-3 in Jecheon had the lowest root dry weight among the 18 cultivation sites, but in terms of active compounds, statistically, the highest content was at 9.0 g/100 g (Table 6, *p* < 0.05). Among the active compounds, the nodakenin content was significantly low in the four sites in PyeongChang, while Je1-3, which had the highest total content, also had the highest levels of nodakenin content (Table 6, *p* < 0.05).

In addition, Je2-1, Je4-1 in the same Jecheon area showed significantly higher values of 1.20 g/100 g to 1.27 g/100 g of nodakenin content. The highest decursin content was in Je1-3 and the lowest was in Je1-1. Decursinolangelates, like the other two compounds, were statistically the highest in the Je1-3 and Je4-2 sites among the total 18 cultivation sites (Table 6, *p* < 0.05). Bon-4 in the Bonghwa area had the lowest statistically significant levels of decursinolangelates among the entire cultivation sites.

Correlation analysis of the altitude, weather conditions, and the active compounds of the root extracts were carried out in Pyeongchang, Jecheon, and Bonghwa regions. It was found that the compound most affected by the weather conditions was nodakenin, which showed a positive correlation with temperature-related factors (Table 7, significant at *p* = 0.01). Nodakenin also showed a negative correlation with altitude, air humidity, root dry weight, the duration of sunshine, and the percentage of sunshine (Table 7, significant at *p* = 0.01). Decursin and ducursinolangelate did not show much relevance with the weather conditions.

In addition, the correlation between the soil characteristics of the sites and the content of active compounds was conducted and all three compounds showed a high degree of correlation with the content of the gravel (Table 8). Nodakenin also showed a positive correlation with soil pH and Na^+^ content (Table 8, significant at *p* = 0.01). Decursinolangelate showed a positive correlation with Ca^2+^ content (Table 8, significant at *p* = 0.05).

## 3. Discussion

Generally, Korean *Angelica* grows naturally in high mountain areas and does not grow well in high-temperature areas. According to studies, areas with an accumulative temperature of less than 3700 °C are suitable for the cultivation site of Korean *Angelica* [22]. Growing Korean *Angelica* in low-altitude areas is not good for the growth due to high-temperature damage caused in summer. In particular, Korean *Angelica* has an advantage in growing in mountainous highlands where there is an average temperature between 20 °C and 22 °C in July and August [1]. In this study, Pyeongchang area showed a high average root mass per individual plant, which was 130.1 g under low-temperature long-day conditions, and in Jecheon (84.4 g) and Bonghwa (100.0 g), where the sites were located below a 500m altitude, and showed a 23 and 35 g/100 g decrease in root mass, respectively, compared to that of Pyeongchang area.

In the recent studies done in Korea, studies on the breeding and cultivation of medicinal plants have been actively conducted but little has been done for active compounds except for [23,24,25]. A study found that ginseng, a major medicinal crop in Korea, has a lower content of ginsenoside in better growth conditions [23]. In addition, due to different weather conditions during the growth period, ginseng grown in the vinyl house tended to have a higher weight of small roots and higher content for certain ginsenosides [24]. In conclusion, although growth was not good in the cultivation sites where the weather condition was not optimal due to a low altitude, the content of nodakenin was high.

Environmental factors, such as light conditions, soil acidity, moisture content, organic matter, nutritional saline content, etc., are very complex and such heterogeneity has a significant effect on plant growth and utility [26,27]. Although studies have been actively conducted on how the growth of herbs changes with these environmental factors [28,29,30], there have not been many studies on the properties of useful ingredients. The active compounds of plants are affected by a variety of factors, including weather, soil, and genetic factors, and each component is synthesized through different paths in different environments [31]. Foreign studies are being conducted to identify optimal reproductive conditions that could increase the specific functional component (index, active component) as well as the biomass of medicinal crops [32,33]. In addition, a medicinal component called the benzopheanthridine alkaloid, which has an antibacterial effect, had a good growth medium, but the component showed a high value in its natural habitat [34]. Furthermore, for medicinal plants native to Canada’s maple forests, research has been published that favorable soil and light conditions for growth increase the total amount of useful ingredients [33]. Coumarin derivatives, including decursin and decursinolangelate, play an important role in the prevention of plant pathogens and in the response mechanism to non-biological [35]. In this study, a lot of gravel in the soil may have caused non-biological stress on this plant. As to the reasons for the above results, more detailed research is needed to pinpoint the factors that increase the content of each component. Cu ion is a well-known nonbiological elicitor and is known to be very effective in inducing the biosynthesis of cumarin compounds, which include decursin and decursinolangelate. The productivity of betacyanin, the same coumarin component, was increased by 1.3 times by the processing of Cu ions in the cell culture of *Portulaca grandiflora* and was reported to induce the production of coumarin compounds even in sunflowers [36]. In recent years, some studies have been conducted to increase the content of decursin and decursinolanegelate [37]. When agents induced with Cu ions were added to the yeast extract and were treated every two weeks during the first 10 weeks and then harvested, decursin and decursinolanegelate increased by about 1.5 to 1.7 times. In these papers, the technique of Cu ion treatment, how Cu ion is absorbed into the cells of the plants, and thus how active compounds are enhanced are explained [36,37].

## 4. Materials and Methods

### 4.1. Study Sites

This study was carried out in 4 to 10 cultivation sites according to the GAP (Great Agricultural Products Certification) cultivation patterns in Pyeongchang, Gangwon Province, Jecheon, Chungcheongbuk-do, and Bonghwa, Kyungsangbukdo Province (Table 9).

The location of each cultivation site was chosen as the study sites have little slope and are not obscured by surrounding trees. After raising seedlings for one year in the open field, they were then transplanted into the cultivation site. The 2-year-old seedlings of middle size, measuring 0.7 to 0.9 centimeters in root diameter, were planted in two rows at a 20-centimeter depth (interval of seedlings: between 25 and 30 centimeters). In Pyeongchang cultivation sites, except for the Pye2-5 being at a 770-meter altitude, the remaining three cultivation sites were at a 674- to 697-meter altitude. In addition, ‘Yeongheung’ variety (No. 05-0032-5) registered in 2008 in the National Seed Resources Agency were transplanted starting from the end of March to early April. The cultivation sites of Jecheon is at 237 to 385-meter altitude. Except for Je1-1, Je3-2, and Je3-3, the seedlings were purchased from the seedling nursery site of ‘Yeongheung’ cultivar in PyeongChang. After a year of sowing seeds from Mt. Bangtae, the seedlings were then transplanted into the cultivation site in Je3-2 and Je3-3 (Table 9). The cultivation sites of Bonghwa were located at 318- to 378-meter altitude. Especially, Bon-2 was located in at the highest altitude among the four cultivation sites. The origin of seedlings in Bon-1, Bon-2, and Bon-3 was different since they were purchased from different places, and the seedlings of ‘manchu’ cultivar developed by the Rural Development Administration were planted in Bon-4 (Table 9).

### 4.2. Soil Analysis

Soil cores within the root zone of the plant were sampled at three locations in the cultivation sites. The soil analysis was commissioned to the Korea Forestry Promotion Institute for all the necessary tests except for the gravel content. The content of gravel was measured by the weight of a gravel component of 2 mm or more when the specimen was dried and sifted using a sieve of 2 mm. The chemical properties of the soil were measured with a specimen that passed through a 2-mm sieve. Soil texture analysis, which can measure the content of sand, mass, and clay in soil, was measured based on Stokes’s law [38]. The organic content in the soil was measured by the weight difference of soil organic matter (g/100 g) after burning the organic matter in the soil immediately after drying at 600 °C for six hours (the soil organic matter content (g/100 g) = [(WBefore − Wafter) / WBefore] × 100). Soil pH was measured using a pH meter and the soil samples were mixed with distilled water at a ratio of 1:5. The nitrogen content was measured using the Micro Kjeldahl Act [39], the Bray No.1 [40], and the cation exchange capacity [41]. The cation content of Ca, K, Mg, and Na was measured using the plasma emission photometric method [41].

### 4.3. Meteorological Data

Data on weather conditions at the cultivation sites were collected from Daegwallyeong, Jecheon and Bonghwa branches, using the Korean Meteorological Administration’s open portal (www.data.kma.go.kr). In addition, the atmospheric temperature and moisture systems (S-TMB-M002, ONset Computer Corporation, Bourne, MA, USA) and soil temperature and humidity measuring devices (S-TMB-002, S-SMA-005M-On) were installed on the two to three cultivation sites from July to September, 2017. However, the Pye2-5 was not included in the analysis as data was unavailable since September 6, 2017. Bonghwa area was evaluated from September 21 to November 2, 2017. According to the data from the Korea Meteorological Administration’s Daegwallyeong branch, the monthly mean temperature of Pyeongchang from April to October 2017, which is the period of the growth of Korean *Angelica*, was 14.7 °C, which is 3.2 to 3.8 °C lower than that of the Jecheon and Bonghwa regions (Table 10).

Furthermore, data showed the months with the highest temperature in the three cultivation sites. Pyeongchang had 31.1 °C in July, while in August, Jecheon and Bonghwa had 36.8 and 34.6 °C respectively (Table 10). The lowest temperature readings were −5.1 °C in October for both Bonghwa and Jecheon while it reached −5.3 °C in Pyeongchang (Table 10). The accumulative temperature based on 5 °C was 3123.3 °C for Pyeongchang, the lowest among the three regions, and Jecheon had the highest temperature of 3959.5°C (Table 10). The average dew point temperature was 6.1 °C for Pyeongchang, the lowest among the three regions, and 8.1 °C for Bonghwa (Table 10). The monthly mean relative humidity was 85.0%, the highest among the three regions in Pyeongchang, and the lowest in Jecheon at 74.0%. The total precipitation was the highest at 948.6 mm in Jecheon, and the lowest at 725.2 mm in Bonghwa (Table 10). In contrast to the temperature, Pyeongchang had the longest duration of sunshine exposure, with a mean percentage of 59.7% (Table 10).

### 4.4. Analysis of the Growth Characteristics

During the optimum harvest time, between 10 and 36 of the samples were collected from four cultivation sites in Pyeongchang, 10 cultivation sites in Jecheon, and 4 cultivation sites in Bonghwa between October 11 and 13. After the fresh weight of the root and the shoot were measured, they were dried at 35 to 40 °C for 10 days until there was no change in weight in the method that growers use and then the final weight for the dry one was measured.

### 4.5. Analysis of Active Compounds

**Figure 1:**
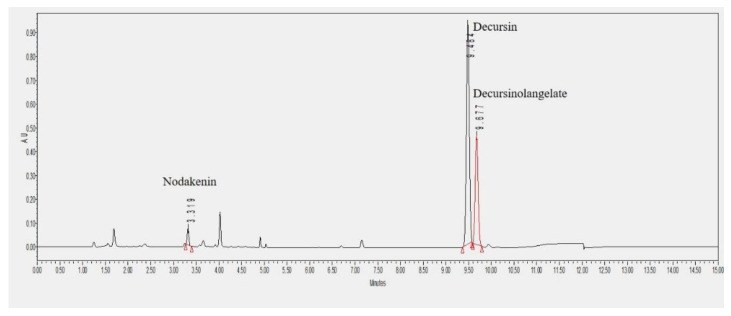
UPLC chromatogram of indicator components for the *Angelica gigas* samples.

Each sample was labeled and then crushed, and 10 mg was extracted using 1 mL of 100% MeOH for one hour. Extracts were centrifuged for 20 min at 3000 rpm and filtered with a 0.2-μm membrane filter (Whatman PTFE Syringe Filter, UK) for analysis. The content analysis was conducted using decursin, decursinolangelate, and nodakenin, which are the indicator components of the *Korean Angelica* as standard substances purchased from the National Institute for Korean Medicine Development and had a purity of 97% or more. MeOH, acetonitrile, formic acid, and water, used in extraction and UPLC analysis, was purchased from J. T. Baker (USA) products, and formic acid used was from Sigma-Aldrich (USA) products. Analysis of the standard substances and extracts of samples were analyzed using the Waters Acuity I-class UPLC system (Waters, USA), and the HPLC conditions shown in the Korean Pharmaceuticals were modified to establish the optimal analysis method for UPLC conditions. The column used was the Waters Acquity BEH C18 column (1.7 μm, 2.1 × 100 mm) and the temperature of the column was maintained at 35 °C. The mobile phase started with a ratio of solution A (0.1% formic acid in water): Solution B (acetonitrile containing 0.1% formic acid). The ratio was started to 80:20 →1 min, 70:30→ 3 min, 50:50 → 3.5 min, 45:55 → 9 min, 0:100→ 9.5 min, 0:100→ 10.5 min, and was finished to 80:20 → 12 min. The sample injection volume was 2 μL, and the PDA detector was measured at UV 330 nm. Based on the analysis conditions established, the three compounds nodakenin, decursin, and decursinolangelate were analyzed for six concentrations (200, 100, 50, 25, 12.5, and 6.25 µg/mL) and each calibration curve was prepared. The calibration coefficient value of the calibration curve was determined, and the contents of each compound were calculated (Table 11, r^2^ ≥ 0.997). The peak width of each compound was measured by analyzing each chromatogram obtained through the above analysis method through Empower Software 2.0. The measured width was calculated in mg/mL using the expression of the calibration curve (Figure 1).

### 4.6. Data Analysis

The soil and growth characteristics of the cultivation sites were not divided into regions, and MANOVA was carried out using the SPSS 20.0 Program (IBM Corporation, v. 20.0 Armonk, NY, USA) to determine if the total 18 cultivation sites in Pyeongchang, Jecheon, and Bonghwa showed statistically significant differences, and the follow-up test Duncan′s test was used. The active compounds were averaged using the data analyzed three times per specimen of an object, and statistical processing was performed in a manner similar to the evaluation of the plant growth characteristics. A correlation analysis was performed to identify the link between the environmental characteristics, the plant growth characteristics, and active compounds, and the Pearson correlation coefficient was used for the analysis (SPSS v. 20.0).

## 5. Conclusions

The root dry weight from the cultivation site increased significantly when the conditions of the environment were similar to the natural habitat. Pyeongchang, which has a high root weight, was located in a 674~770 m altitude with an accumulative temperature of 3123 °C and an organic matter content of 6.9 ± 1.9%, total N content of 0.4 ± 0%, 1121.3 ± 382.0 mg^−1^ kg^−1^ of available P_2_O_5_ content, and CEC of 14.81 ± 2.6 cmolc kg^−1^. Correlation analysis of active compounds showed that decursin was high in groups with a high content of soil gravel. Decursinolangelates showed the highest correlation with the content of soil gravel in the site, while nodakenin was high in the environments with high annual average temperatures and low solar hours, which was during the time period of April to October. Currently, cultivation sites show less than 6.0 g/100 g of active compounds due to the weather conditions of the year and the environmental condition of the site; therefore, the domestic production of Korean *Angelica* is often not used as a raw material for functional food. This study concludes that the root yield in the cultivation sites would improve if weather conditions are maintained at a similar level as in their natural habitat. Additionally, the environment that improves root growth did not increase the content of active compounds, and when there was a high gravel content and high temperature during the growth period, the content of active compounds increased.

## Figures and Tables

**Table 1:** Soil textures in *Angelica gigas* cultivation sites, Korea (mean ± SD).

Region	CultivationSites	The Content of Gravel (%)	The Content of Sand (%)	The Content of Silt (%)	The Content of Clay (%)
Pyeongchang	Pye1-1	32.7 ± 5.0 ^b^	50.4 ± 16.3 ^b^	35.1 ± 12.0 ^b^	14.5 ± 4.8 ^a^
	Pye1-2	30.0 ± 1.2 ^b^	54.2 ± 2.9 ^b^	32.1 ± 2.5 ^b^	13.7 ± 0.7 ^b^
	Pye2-1	38.9 ± 4.3 ^b^	71.8 ± 5.6 ^a^	19.5 ± 4.6 ^c^	8.7 ± 1.1 ^b^
	Pye2-5	33.3 ± 3.2 ^b^	58.4 ± 3.7 ^b^	30.1 ± 1.9 ^b^	11.5 ± 2.1 ^b^
	Mean	–	58.7 ± 5.4	29.2 ± 3.2	12.1 ± 2.6
Jecheon	Je1-1	43.2 ± 2.1 ^b^	46.9 ± 28.9 ^c^	41.2 ± 27.6 ^a^	11.9 ± 2.5 ^b^
	Je1-2	50.4 ± 6.6 ^b^	61.9 ± 3.5 ^b^	28.8 ± 3.4 ^b^	9.3 ± 0.7 ^b^
	Je1-3	62.0 ± 3.3 ^a^	58.7 ± 2.4 ^b^	28.4 ± 2.3 ^b^	12.9 ± 0.2 ^b^
	Je1-4	38.4 ± 5.1^b^	65.9 ± 1.3 ^b^	25.4 ± 1.1 ^b^	8.7 ± 0.2 ^b^
	Je2-1	35.7 ± 3.1 ^b^	68.8 ± 3.5 ^b^	24.1 ± 2.8 ^b^	7.1 ± 0.8 ^b^
	Je3-1	53.3 ± 2.1 ^b^	52.4 ± 4.3 ^b^	35.7 ± 3.4 ^b^	12.0 ± 1.2 ^b^
	Je3-2	40.9 ± 3.1 ^b^	56.5 ± 0.5 ^b^	34.1 ± 0.2 ^b^	9.4 ± 0.4 ^b^
	Je3-3	48.0 ± 6.0 ^b^	57.4 ± 2.5 ^b^	31.8 ± 1.6 ^b^	10.8 ± 1.2 ^b^
	Je4-1	–	66.9 ± 0.7 ^b^	24.4 ± 0.8 ^b^	8.7 ± 0.3 ^b^
	Je4-2	–	71.0 ± 1.2 ^b^	20.6 ± 0.8 ^b^	8.3 ± 1.2 ^b^
	Mean	–	60.6 ± 11.9	29.5 ± 10.8	9.9 ± 2.1
Bonghwa	Bon-1	35.2 ± 2.0 ^b^	68.8 ± 4.7 ^b^	22.3 ± 3.3 ^b^	8.9 ± 1.6 ^b^
	Bon-2	44.4 ± 5.0 ^b^	64.6 ± 4.4 ^b^	24.4 ± 2.8 ^b^	11.0 ± 1.7 ^b^
	Bon-3	26.0 ± 3.1 ^c^	71.8 ± 0.6 ^b^	23.1 ± 0.7 ^b^	5.0 ± 0.1 ^c^
	Bon-4	–	75.2 ± 1.6 ^a^	18.4 ± 1.4 ^c^	6.4 ± 0.3 ^c^
	Mean	–	70.1 ± 12.1	22.1 ± 8.8	7.8 ± 3.5

* a, b, c: value in same columns with different superscripts are significantly different (*p* < 0.05) with one way ANOVA and Duncan’s test (alpha = 0.05) using SPSS statistics (IBM Corporation, Ver. 20.0 Armonk, NY, USA).

**Table 2:** Edaphic characteristics of *Angelica gigas* cultivation sites, Korea.

Region	Cultivated Sites	pH (1:5, H2O)	Organic Matter (g/100g)	Total N (g/100g)	Available P_2_O_5_ (mg/kg)	CEC (cmolc kg^−1^)	K^+^	Na^+^	Ca^2+^	Mg^2+^
									cmolc kg^-1^	
**Pyeongchang**	Pye1-1	4.6 ± 0.2 ^c^	6.4 ± 2.9 ^a^	0.4 ± 0.1 ^a^	990 ± 38 ^b^	13.8 ± 4.2 ^b^	0.9 ± 0.3 ^b^	0.1 ± 0.0 ^b^	4.1 ± 0.9 ^b^	0.8 ± 0.2 ^b^
	Pye1-2	5.0 ± 0.2 ^b^	6.9 ± 0.3 ^a^	0.4 ± 0.0 ^a^	1121 ± 124 ^b^	14.8 ± 0.6 ^a^	0.9 ± 0.2 ^b^	0.1 ± 0.0 ^b^	4.1 ± 0.6 ^b^	0.6 ± 0.1 ^b^
	Pye2-1	4.4 ± 0.6 ^b^	3.9 ± 0.8 ^b^	0.3 ± 0.1 ^b^	1456 ± 57 ^a^	10.3 ± 0.8 ^b^	0.7 ± 0.1 ^b^	0.1 ± 0.0 ^b^	2.7 ± 0.2 ^b^	0.3 ± 0.1 ^c^
	Pye2-5	4.2 ± 0.2 ^c^	6.0 ± 0.7 ^a^	0.4 ± 0.0 ^b^	1018 ± 235 ^b^	12.9 ± 1.0 ^a^	0.6 ± 0.1 ^b^	0.1 ± 0.0 ^b^	2.3 ± 0.5 ^c^	0.4 ± 0.2 ^b^
	Mean	4.6 ± 0.5	5.8 ± 1.9	0.4 ± 0.1	1146 ± 231	13.0 ± 2.8	0.8 ± 0.2	0.1 ± 0.0	3.3 ± 1.0	0.5 ± 0.2
Jecheon	Je1-1	5.0 ± 0.2 ^b^	1.0 ± 0.2 ^b^	0.1 ± 0.0 ^b^	423 ± 213 ^b^	8.1 ± 0.9 ^b^	0.3 ± 0.0 ^b^	0.1 ± 0.0 ^b^	2.3 ± 0.5 ^c^	0.7 ± 0.2 ^b^
	Je1-2	5.9 ± 0.1 ^b^	3.6 ± 0.4 ^b^	0.3 ± 0.0 ^b^	977 ± 30 ^b^	11.9 ± 0.2 ^b^	0.7 ± 0.2 ^b^	0.1 ± 0.0 ^b^	4.7 ± 0.5 ^b^	0.7 ± 0.0 ^b^
	Je1-3	6.6 ± 0.5 ^b^	2.3 ± 0.3 ^b^	0.2 ± 0.0 ^b^	419 ± 34 ^c^	10.0 ± 0.5 ^b^	0.4 ± 0.1 ^b^	0.1 ± 0.0 ^a^	5.5 ± 0.9 ^b^	0.4 ± 0.1 ^b^
	Je1-4	6.3 ± 0.7 ^b^	2.5 ± 0.7 ^b^	0.2 ± 0.0 ^b^	670 ± 115 ^b^	9.0 ± 1.2 ^b^	0.5 ± 0.1 ^b^	0.1 ± 0.0 ^b^	4.6 ± 1.6 ^b^	0.4 ± 0.2 ^b^
	Je2-1	6.3 ± 0.2 ^b^	2.6 ± 0.3 ^b^	0.2 ± 0.0 ^b^	991 ± 56 ^b^	10.9 ± 0.5 ^b^	1.0 ± 0.1 ^a^	0.1 ± 0.0 ^b^	4.6 ± 0.2 ^b^	0.9 ± 0.1 ^b^
	Je3-1	5.5 ± 0.4 ^b^	3.7 ± 0.8 ^b^	0.3 ± 0.0 ^b^	953 ± 83 ^b^	12.3 ± 1.5 ^a^	0.8 ± 0.1 ^b^	0.1 ± 0.0 ^b^	3.3 ± 0.8 ^b^	1.1 ± 0.3 ^b^
	Je3-2	6.4 ± 0.2 ^b^	2.9 ± 0.3 ^b^	0.2 ± 0.0 ^b^	900 ± 60 ^b^	12.4 ± 0.3 ^b^	0.9 ± 0.0 ^b^	0.1 ± 0.0 ^b^	5.7 ± 0.2 ^b^	2.4 ± 0.2 ^a^
	Je3-3	5.2 ± 0.1 ^b^	3.8 ± 0.4 ^b^	0.3 ± 0.0 ^b^	999 ± 174 ^b^	11.6 ± 0.4 ^b^	0.8 ± 0.0 ^b^	0.1 ± 0.0 ^b^	2.6 ± 0.6 ^b^	0.6 ± 0.2 ^b^
	Je4-1	6.7 ± 0.3 ^b^	3.0 ± 0.3 ^b^	0.2 ± 0.0 ^b^	368 ± 129 ^c^	9.2 ± 0.6 ^b^	0.1 ± 0.0 ^c^	0.1 ± 0.0 ^b^	4.4 ± 0.6 ^b^	1.4 ± 0.3 ^b^
	Je4-2	7.7 ± 0.2 ^a^	3.9 ± 0.5 ^b^	0.3 ± 0.0 ^b^	669 ± 65 ^b^	9.3 ± 0.1 ^b^	0.2 ± 0.0 ^b^	0.1 ± 0.0 ^b^	10.4 ± 1.9 ^a^	0.8 ± 0.1 ^b^
	Mean	6.2 ± 0.8	2.9 ± 1.0	0.3 ± 0.0	737 ± 271	10.5 ± 1.6	0.6 ± 0.3	0.1 ± 0.0	4.8 ± 2.3	0.9 ± 0.6
Bonghwa	Bon-1	6.1 ± 0.4 ^b^	0.5 ± 0.0 ^c^	0.1 ± 0.0 ^c^	248 ± 47 ^c^	5.4 ± 0.4 ^c^	0.3 ± 0.0 ^b^	0.0 ± 0.0 ^c^	4.0 ± 0.1 ^b^	0.8 ± 0.0 ^b^
	Bon-2	6.5 ± 0.2 ^b^	1.5 ± 0.1 ^b^	0.2 ± 0.0 ^b^	415 ± 33 ^c^	7.2 ± 0.8 ^b^	0.5 ± 0.1 ^b^	0.1 ± 0.0 ^b^	3.3 ± 0.1 ^b^	0.6 ± 0.1 ^b^
	Bon-3	6.6 ± 0.2 ^b^	3.2 ± 0.2 ^b^	0.3 ± 0.0 ^b^	165 ± 50 ^c^	10.8 ± 0.4 ^b^	0.9 ± 0.1 ^b^	0.1 ± 0.0 ^b^	5.1 ± 0.3 ^b^	1.1 ± 0.1 ^b^
	Bon-4	6.9 ± 0.2 ^b^	1.5 ± 0.2 ^b^	0.2 ± 0.0 ^b^	953 ± 9 ^b^	9.2 ± 0.6 ^b^	0.2 ± 0.0 ^b^	0.1 ± 0.0 ^b^	5.4 ± 0.1 ^b^	1.2 ± 0.1 ^b^
	Mean	6.4 ± 0.4	1.7 ± 1.0	0.2 ± 0.0	770 ± 547	7.8 ± 2.1	0.5 ± 0.3	0.1 ± 0.0	4.4 ± 0.9	0.9 ± 0.2

* a, b, c: value in same columns with different superscripts are significantly different (*p* < 0.05) with one-way ANOVA and Duncan’s test (alpha = 0.05) using SPSS statistics (IBM Corporation, v. 20.0 Armonk, NY, USA).

**Table 3:** Growth characteristics of roots in *Angelica gigas* cultivation sites, Korea (mean ± SD).

Region	Cultivation Sites	Fresh Weight of Shoot (g)	Fresh Weight of Root (g)	Dry Weight of Root (g)	Percent of Water Content in Root (%)	Root/Shoot Ratio
Pyeong chang	Pye1-1	243.8 ± 201.2^b^	495.2 ± 259.4 ^b^	142.4 ± 81.2 ^b^	70.4 ± 12.0 ^b^	2.6 ± 1.1 ^b^
	Pye1-2	169.0 ± 102.0 ^b^	623.9 ± 417.0 ^a^	167.9 ± 138.5 ^a^	68.2 ± 22.6 ^b^	3.8 ± 1.4 ^b^
	Pye2-1	133.1 ± 96.3 ^b^	374.7 ± 207.3 ^b^	100.4 ± 61.0 ^b^	69.3 ± 19.8 ^b^	3.8 ± 2.5 ^b^
	Pye2-5	131.7 ± 92.0 ^b^	541.3 ± 285.8 ^b^	148.4 ± 91.0 ^b^	72.4 ± 5.9 ^b^	5.3 ± 2.5 ^a^
	Mean	176.6 ± 151.5	467.5 ± 275.6	130.1 ± 86.9	70.0 ± 16.0	3.6 ± 2.2
Jecheon	Je1-1	140.8 ± 84.7 ^b^	171.4 ± 91.4 ^c^	43.9 ± 25.0 ^b^	72.2 ± 6.0 ^b^	1.3 ± 0.5 ^c^
	Je1-2	123.0 ± 47.9 ^b^	270.0 ± 166.4 ^b^	87.8 ± 67.4 ^b^	69.4 ± 3.9 ^b^	2.3 ± 1.3 ^b^
	Je1-3	61.0 ± 23.1 ^c^	131.0 ± 62.4 ^c^	34.7 ± 17.4 ^c^	73.4 ± 3.2 ^b^	2.2 ± 0.8 ^b^
	Je1-4	145.5 ± 190.1 ^b^	250.5 ± 106.6 ^b^	69.6 ± 36.4 ^b^	73.1 ± 3.1 ^b^	4.1 ± 3.6 ^b^
	Je2-1	78.0 ± 46.1 ^b^	201.0 ± 158.5 ^b^	35.0 ± 33.2 ^c^	79.1 ± 6.8 ^a^	3.1 ± 2.8 ^b^
	Je3-1	179.8 ± 89.7 ^b^	280.9 ± 119.8 ^b^	69.8 ± 32.8 ^b^	75.4 ± 3.6 ^b^	1.8 ± 0.8 ^b^
	Je3-2	217.7 ± 122.7 ^b^	485.5 ± 290.5 ^b^	149.4 ± 94.7 ^b^	67.5 ± 20.2 ^b^	2.5 ± 1.5 ^b^
	Je3-3	168.6 ± 117.6 ^b^	397.5 ± 241.5 ^b^	115.5 ± 85.6 ^b^	72.5 ± 3.4 ^b^	2.7 ± 1.6 ^b^
	Je4-1	110.5 ± 28.2 ^b^	288.5 ± 136.2 ^b^	84.3 ± 42.9 ^b^	71.2 ± 2.2 ^b^	2.8 ± 1.7 ^b^
	Je4-2	70.3 ± 85.8 ^c^	104.9 ± 49.9 ^c^	33.5 ± 19.1 ^c^	69.1 ± 5.0 ^b^	2.3 ± 1.0 ^b^
	Mean	151.4 ± 111.3	293.9 ± 218.6	84.4 ± 71.9	71.9 ± 9.7	2.3 ± 1.7
Bonghwa	Bon-1	213.3 ± 72.0 ^b^	345.6 ± 153.2 ^b^	106.6 ± 48.8 ^b^	48.6 ± 23.7 ^c^	1.7 ± 0.7 ^b^
	Bon-2	124.4 ± 76.0 ^b^	383.8 ± 129.1 ^b^	96.2 ± 28.3 ^b^	73.0 ± 10.5 ^b^	5.1 ± 3.8 ^b^
	Bon-3	283.0 ± 163.5 ^b^	346.0 ± 139.2 ^b^	107.8 ± 59.9 ^b^	46.0 ± 61.1 ^b^	1.7 ± 1.3 ^b^
	Bon-4	315.0 ± 186.7^a^	361.1 ± 144.8 ^b^	88.2 ± 34.7 ^b^	68.5 ± 8.5 ^b^	1.3 ± 0.3 ^c^
	Mean	238.3 ± 154.2	358.1 ± 136.8	100.0 ± 44.4	58.3 ± 35.6	2.3 ± 2.5

a, b, c: value in same columns with different superscripts are significantly different (*p* < 0.05) with oneway. ANOVA and Duncan’s test (alpha = 0.05) using SPSS statistics (IBM Corporation, v. 20.0 Armonk, NY, USA).

**Table 4:** Correlation coefficients between root growth characteristics and meteorological factors in *Angelica gigas* Nakai cultivation sites, Korea.

Meteorological Factor	Dry Weight of Root	Percent of Water Content in Root (%)	Root/Shoot Ratio
Altitude (m)	0.646 **	0.187	0.608 **
Mean temperature (℃)	−0.645 **	−0.091	−0.498 *
Mean minimum temperature (℃)	−0.627 **	−0.139	−0.463
Mean maximum temperature (℃)	−0.659 **	−0.013	−0.497 *
The highest temperature (℃)	−0.604 **	−0.185	−0.502 *
The lowest temperature (℃)	−0.650 **	0.093	−0.412
The mean temperature of dew point (℃)	−0.624 **	−0.147	−0.503 *
Mean Air humidity (g/100g)	0.659 **	0.003	0.467 *
Annual precipitation (mm)	−0.094	0.486 *	0.142
The duration of sunshine (hr)	0.547*	0.264	0.498 *
The percentage of sunshine (g/100g)	0.542*	0.269	0.482
The minimum grass temperature (℃)	−0.613 **	0.192	−0.354
Dry weight of root	1.000	−0.112	0.132
Percent of water content in root	−0.112	1.000	−0.182
Root/shoot ratio	0.132	−0.183	1

*Significant at *p* = 0.05, ** Significant at *p* = 0.01.

**Table 5:** Correlation coefficients between root growth characteristics and edaphic characteristics in *Angelica gigas* Nakai cultivation sites, Korea.

Edaphic Characteristics	Dry Weight of Root	Percent of Water Content in Root	Root/Shoot Ratio
pH	−0.514 *	−0.185	−0.34
Gravel content	−0.282	0.254	−0.232
Organic matter	0.590 **	0.376	0.39
Total N	0.506*	0.386	0.38
Available P_2_O_5_	0.386	0.387	0.67
CEC	0.537*	0.568*	0.18
The content of sand	0.148	0.304	0.43
The content of silt	−0.156	−0.241	−0.43
The content of clay	−0.092	−0.396	−0.34
K^+^	0.037	−0.017	0.00
Na^+^	−0.560 *	−0.054	0.00
Ca^2+^	−0.239	−0.111	0.20
Mg^2+^	0.300	0.026	0.09

*Significant at *p* = 0.05, ** Significant at *p* = 0.01.

**Table 6:** Active compounds in root extracts of *Angelica gigas* Nakai cultivation sites, Korea (mean ± SD).

Region	Cultivation Sites	Total Active Compounds (g/100 g)	Nodakenin (g/100 g)	Decursin (g/100 g)	Decursinol Angelate (g/100 g)
Pyeongchang	Pye1-1	5.27 ± 1.31 ^c^	0.30 ± 0.09 ^c^	2.90 ± 0.83 ^b^	2.07 ± 0.57 ^b^
	Pye1-2	5.62 ± 1.46 ^b^	0.29 ± 0.07 ^c^	3.22 ± 0.77 ^b^	2.11 ± 0.68 ^b^
	Pye2-1	5.87 ± 1.65 ^b^	0.35 ± 0.09 ^c^	3.18 ± 0.97 ^b^	2.34 ± 0.68 ^b^
	Pye2-5	5.89 ± 1.06 ^b^	0.29 ± 0.10 ^c^	3.22 ± 0.65 ^b^	2.38 ± 0.50 ^b^
	Mean	5.63 ± 1.46	0.31 ± 0.10	3.09 ± 0.87	2.22 ± 0.63
Jecheon	Je1-1	5.28 ± 1.38^c^	0.76 ± 0.24 ^b^	2.49 ± 0.70 ^c^	2.03 ± 0.57 ^b^
	Je1-2	6.50 ± 0.79 ^b^	0.89 ± 0.21 ^b^	3.27 ± 0.46 ^b^	2.34 ± 0.31 ^b^
	Je1-3	9.03 ± 2.33^a^	1.18 ± 0.29 ^a^	4.42 ± 1.09 ^a^	3.43 ± 1.20 ^a^
	Je1-4	5.82 ± 1.16 ^b^	0.89 ± 0.14 ^b^	2.66 ± 0.55 ^b^	2.26 ± 0.57 ^b^
	Je2-1	7.07 ± 1.73 ^b^	1.27 ± 0.26 ^a^	3.17 ± 1.17 ^b^	2.63 ± 0.71 ^b^
	Je3-1	7.61 ± 1.80 ^b^	0.92 ± 0.26 ^b^	4.12 ± 0.91 ^b^	2.57 ± 0.78 ^b^
	Je3-2	7.27 ± 1.85 ^b^	0.95 ± 0.27 ^b^	3.89 ± 0.94 ^b^	2.43 ± 0.87 ^b^
	Je3-3	7.84 ± 2.14 ^b^	0.90 ± 0.24 ^b^	3.90 ± 1.14 ^b^	3.03 ± 0.90 ^b^
	Je4-1	6.31 ± 1.55 ^b^	1.20 ± 0.38 ^a^	2.79 ± 0.79 ^b^	2.32 ± 0.61 ^b^
	Je4-2	7.80 ± 3.54 ^b^	1.01 ± 0.55 ^b^	3.64 ± 1.71 ^b^	3.15 ± 1.34 ^a^
	Mean	7.02 ± 2.18	0.94 ± 0.31	3.50 ± 1.71	2.58 ± 0.91
Bonghwa	Bon-1	6.24 ± 1.64 ^b^	0.64 ± 0.12 ^b^	3.22 ± 0.82 ^b^	2.37 ± 0.79 ^b^
	Bon-2	6.40 ± 1.11 ^b^	0.88 ± 0.28 ^b^	3.46 ± 0.65 ^b^	2.06 ± 0.39 ^b^
	Bon-3	6.14 ± 1.31 ^b^	0.76 ± 0.19 ^b^	3.27 ± 1.01 ^b^	2.10 ± 0.45 ^b^
	Bon-4	5.92 ± 1.27 ^b^	0.75 ± 0.11 ^b^	3.24 ± 0.69 ^b^	1.93 ± 0.64 ^c^
	Mean	6.17 ± 1.36	0.76 ± 0.20	3.29 ± 0.82	2.12 ± 0.61

a, b, c: value in same columns with different superscripts are significantly different (*p* < 0.05) with oneway ANOVA and Duncan’s test (alpha = 0.05) using SPSS statistics (IBMCorporation, Ver. 20.0 Armonk, NY, USA).

**Table 7:** Correlation coefficients between active compounds and meteorological factors in *Angelica gigas* Nakai cultivation sites, Korea.

Meteorological Factors	Total Content of Active Compounds (g/100 g)	Nodakenin (g/100 g)	Decursin (g/100 g)	Decursinol Angelate (g/100 g)
Altitude	−0.403	−0.810 **	−0.141	−0.209
Mean temperature	0.533 *	0.896 **	0.242	0.341
Mean minimum temperature	0.568 *	0.911 **	0.250	0.408
Mean maximum temperature	0.503 *	0.875 **	0.233	0.293
The highest temperature	0.468	0.845 **	0.223	0.244
The lowest temperature	0.594 **	0.894 **	0.251	0.483 *
The mean temperature of dew point	0.497 *	0.871 **	0.232	0.286
Air humidity	−0.574 *	−0.912 **	−0.251	−0.421
Annual precipitation	0.230	0.104	0.055	0.426
The duration of sunshine	−0.394	−0.769 **	−0.197	−0.148
The percentage of sunshine	−0.389	−0.763 **	−0.196	−0.141
The minimum grass temperature	0.591 **	0.838 **	0.241	0.534 *
Dry weight of root	−0.437	−0.727 **	−0.076	−0.439
Percent of water content in root	0.126	0.160	0.106	0.067
Total content of active compounds	1.000	0.659 **	0.874 **	0.905 **
Nodakenin	0.659 **	1.000	0.310	0.494 *
Decursin	0.874 **	0.310	1.000	0.705 **
Decursinol angelate	0.905 **	0.494 *	0.705 **	1.000

*Significant at *p* = 0.05, ** Significant at *p* = 0.01.

**Table 8:** Correlation coefficients between active compounds and edaphic characteristics in *Angelica gigas* Nakai cultivation sites, Korea.

Edaphic Characteristics	Total Active Compounds (g/100 g)	Nodakenin (g/100 g)	Decursin (g/100 g)	Decursinol Angelate (g/100 g)
pH	0.455	0.744 **	0.232	0.278
Gravel content	0.738 **	0.575 *	0.631 *	0.684 **
Organic matter	−0.166	−0.563 *	0.018	0.003
Total N	−0.123	−0.545 *	0.034	0.068
Available P_2_O_5_	−0.183	−0.414	0.041	−0.191
CEC	−0.010	−0.356	0.191	0.014
The content of sand	−0.151	−0.239	−0.196	0.046
The content of silt	0.117	0.241	0.142	−0.067
The content of clay	0.205	0.184	0.287	0.014
K^+^	0.133	0.073	0.289	−0.082
Na^+^	0.430	0.714 **	0.140	0.350
Ca^2+^	0.400	0.199	0.239	0.550 *
Mg^2+^	0.078	0.040	0.133	0.004
EC	−0.224	−0.168	−0.110	−0.298
NaCl	−0.177	−0.447	−0.111	0.036
Dry weight of root	−0.437	−0.727 **	−0.076	−0.439
Percent of water content in root	0.126	0.160	0.106	0.067
Total active compounds	1.000	0.659 **	0.874 **	0.905 **
Nodakenin	0.659 **	1.000	0.310	0.494
Decursin	0.874 **	0.310	1.000	0.705 **
Decursinol angelate	0.905 **	0.494 *	0.705 **	1.000

* Significant at *p* = 0.05, ** Significant at *p* = 0.01.

**Table 9:** *Angelica gigas* Nakai cultivation sites on 2017 in Pyeongchang, Jecheon, and Bonghwa, Korea.

Region	Province	Grower	Cultivation Sites		Origin	Altitude (m)
Pyeongchang	Sangjinbu-ri	A	Pye1-1	Young Heung		674
	Sangjinbu-ri	A	Pye1-2	Young Heung		674
	Tapdong-ri	B	Pye2-1	Young Heung		697
	Tapdong-ri	C	Pye2-5	Young Heung		770
Jecheon	Wonwol-ri	D	Je1-1	Bonghwa gun		237
	Wonwol-ri	E	Je1-2	Young Heung		320
	Wonwol-ri	F	Je1-3	Young Heung		314
	Wonwol-ri	F	Je1-4	Young Heung		306
	Deokdong-ri	G	Je2-1	Young Heung		385
	Pojeon-ri	H	Je3-1	–		287
	Omi-ri	I	Je3-2	Mt. Bangtae		363
	Omi-ri	I	Je3-3	Mt. Bangtae		370
	Banghak-ri	J	Je4-1	Young Heung		288
	Wonwol-ri	K	Je4-2	Young Heung		299
Bonghwa	Buncheon-ri	M	Bon-1	–		334
	Buncheon-ri	N	Bon-2	–		378
	Hyeondong-ri	O	Bon-3	–		318
	Hyeondong-ri	M	Bon-4	Manchu		320

**Table 10:** Meteorological characteristics of *Angelica gigas* Nakai cultivation sites (data measured in Korea Meteorological Administration from April to October 2017).

Meteorological Characteristics	Pyeongchang	Jecheon	Bonghwa
Mean temperature (℃)	14.7	18.5	17.9
The highest temperature (℃)	31.1 (July)	36.8 (August)	34.6 (August)
The lowest temperature (℃)	−5.3 (October)	−5.1 (October)	−5.1 (October)
Accumulative temperature (℃)	3123.3	3959.5	3810.3
Mean dew point temperature (℃)	6.1	7.5	8.1
Mean relatively humidity (g/100g)	85.0	74.0	78.0
Mean total precipitation (mm)	937.0	948.6	725.2
Sum of sunshine duration (h)	1708.6	1355.9	1271.2
Mean percentage of sunshine (g/100g)	59.7	47.6	44.5

**Table 11:** Calibration curve equations of Nodakenin, Decursin, and Decursinol angelate.

Compounds	Calibration Curve Equation	r^2^
Nodakenin	y = 26063x + 5418	0.999
Decursin	y = 34383x + 65687	0.999
Decursinol angelate	y = 29111x + 25040	0.999

Y and x are the peak area and the concentration of the analytes respectively.
